# Droplet Rolling and Spinning in V-Shaped Hydrophobic Surfaces for Environmental Dust Mitigation

**DOI:** 10.3390/molecules25133039

**Published:** 2020-07-03

**Authors:** Mubarak Yakubu, Bekir Sami Yilbas, Abba A. Abubakr, Hussain Al-Qahtani

**Affiliations:** 1 Mechanical Engineering Department, KFUPM, Dhahran 31261, Saudi Arabia; g201705190@kfupm.edu.sa (M.Y.); abba.abubakar@kfupm.edu.sa (A.A.A.); qahtanih@kfupm.edu.sa (H.A.-Q.); 2 Center of Research Excellence in Renewable Energy (CoRE-RE), KFUPM, Dhahran 31261, Saudi Arabia; 3 Senior Researcher at K.A.CARE Energy Research & Innovation Center, Dhahran 31261, Saudi Arabia

**Keywords:** droplet, rolling, spinning, dust mitigation, hydrophobic surface

## Abstract

The motion of a water droplet in a hydrophobic wedge fixture was examined to assess droplet rolling and spinning for improved dust mitigation from surfaces. A wedge fixture composed of two inclined hydrophobic plates had different wetting states on surfaces. Droplet rolling and spinning velocities were analyzed and findings were compared with the experiments. A wedge fixture was designed and realized using a 3D printing facility and a high speed recording system was adopted to evaluate droplet motion in the wedge fixture. Polycarbonate sheets were used as plates in the fixture, and solution crystallization and functionalized silica particles coating were adopted separately on plate surfaces, which provided different wetting states on each plate surface while generating different droplet pinning forces on each hydrophobic plate surface. This arrangement also gave rise to the spinning of rolling droplets in the wedge fixture. Experiments were extended to include dust mitigation from inclined hydrophobic surfaces while incorporating spinning- and rolling droplet and rolling droplet-only cases. The findings revealed the wedge fixture arrangement resulted in spinning and rolling droplets and spinning velocity became almost 25% of the droplet rolling velocity, which agrees well with both predictions and experiments. Rolling and spinning droplet gave rise to parallel edges droplet paths on dusty hydrophobic surfaces while striations in droplet paths were observed for rolling droplet-only cases. Spinning and rolling droplets mitigated a relatively larger area of dust on inclined hydrophobic surfaces as compared to their counterparts corresponding to rolling droplet-only cases.

## 1. Introduction

The rate of dust storms around the globe is increasing because of climate change. Devices in open environments are exposed to dusty weather conditions for long durations, which gradually degrade device performances. The sustainability of such devices for prolonged operations requires regular removal of dust from open surfaces. In some cases, the process of dust removal, via cleaning, may not be economically viable for long periods. Although several methods are proposed for cleaning in dusty environments, such as filtering, water jet spreading, mechanical brushing, air blowing, and electrostatic repelling [1,2], these methods are energy-intensive and require additional power and/or clean water sources to accomplish the cleaning process. An efficient cleaning process becomes challenging in terms of cost and maintaining regularity of the process particularly for large areas subjected to cleaning, such as solar energy harvesting farms. One of the methods that ease the cleaning process, in terms of lost cost and minimum external power requirements, is self-cleaning of surfaces while mimicking nature. Self-cleaning surfaces mainly utilize water droplet mobility picking up dust from hydrophobic surfaces under gravitational potential [3]. Surface texturing and low surface free energy of materials becomes important for achieving hydrophobic state on surfaces [4]. Surface texture with hieratically distributed micro/nano pillars is favorable for realizing hydrophobicity on surfaces. However, low contact angle hysteresis becomes crucial for reducing droplet pinning on hydrophobic surfaces, which necessitates the presence of nanoscale stubble-like structures distributed over textured surfaces. Nanoscale stubble-like structures can create a lotus effect on surfaces while reducing interfacial tension of droplet liquid on the wetted surface. Increasing droplet pinning causes sliding and rolling of droplet motion on hydrophobic surfaces, which alters droplet dynamics and the amount of dust mitigation from surfaces. In general, dust mitigation from hydrophobic surfaces by rolling and sliding droplets form one of the fundamental bases for the self-cleaning process [5,6]. The efforts required to remove wetted and dried dust particles from the surface become extremely large [6]. In general, dust particles can be mitigated by rolling liquid droplets along their path [6]. Increasing droplet volume enlarges the width of the droplet path on surfaces; therefore, the area cleaned becomes larger for larger droplet sizes. However, as droplet size increases, wobbling is resulted because of the gravitational potential [4]. Hence, droplet puddles and puddling increases with enlarging droplet size. This gives rise to waviness in the droplet path (striation like edges) while lowering the dust removal area on surfaces. Although striation-like path edges disappear for small size droplets in the droplet path because of small droplet puddling, the droplet path width on surfaces remains narrow and the dusty area cleaned by rolling droplet becomes small. One of the alternative methods reducing striation-like edges on the droplet path for large droplets is spinning of droplet while the droplet is rolling on hydrophobic surfaces. To generate such spinning, change of angular momentum on the rolling droplet needs to be generated. Therefore, investigation of mechanism of spinning for rolling droplet on hydrophobic surface becomes fruitful for dust mitigation from surfaces.

Dust settlement on surfaces is one of the major concerns for the sustainable performance of photovoltaic devices. This is mainly because of lowering surface transmittance of photovoltaic panels and increasing panel temperature through solar absorption despite the fact, that wind blow over panels creates convection cooling and assists mitigation of some dust from surfaces [7]. Dust influence on panel surfaces mostly depends on dust shapes, size distributions, deposition rates, and mineral compositions. However, weather ambient conditions such as wind and humidity influences the soiling of panel surfaces via dust settlements. This becomes critical in humid air ambient since dew driven soiling results in strong adhesion of dust layer on surfaces and wind-assisted dust mitigation becomes almost impossible [8]. Soiling of panel surfaces causes large panel power loss by prolonged dust exposure durations. For concentrated solar power applications, dust soling causes optical reflectance losses, which signifies in humid ambient conditions. This is because of mud formed on surfaces at high humid air conditions and mud drying: Much work is required to remove adhered dry mud and the oxyhydroxide metal compounds formed reduce surface reflectively considerably [9]. Hence, dust mitigation from solar harvesting devices becomes a necessity rather than preference. Self-cleaning of surfaces towards dust mitigation remains important because of cost-effective handling of the cleaning process. Fabrication of hydrophobic surfaces while mimicking nature is one of the alternative methods creating dust repelling surfaces, such as hydrophobizing, via mimicking eucalyptus wood surfaces, by perfluoroalkyltrichlorosilane (PFDS) treatment [10]. In some cases, antireflection of optical rays from surfaces becomes desirable, such as trough surfaces used for solar power harnessing. Generating hydrophobic states on such surfaces is challenging because of the difficulties in the attachment of grafted small hydrophobic molecules on surfaces during coating process. Introducing fluoropolymer brush grafted silica particles in coating overcomes this difficulties and achievement of a durable hydrophobic state on antireflecting surfaces becomes possible [11,12]. Although several methods can be used to create hydrophobic wetting on a surface for self-cleaning applications, a one-step process for achieving hydrophobic wetting state is always demanding [13].

Rolling/sliding water droplets on hydrophobic surfaces can be utilized for dust mitigation from surfaces. To identify texture characteristics of the hydrophobic surface, Bond and Weber numbers play a major role in the mode of droplet motion on surfaces. For large contact angles and high Bond and Weber numbers, the droplet rolls on surfaces; alternatively, droplet sliding dominates over droplet rolling [14]. As droplet inertia and weight forces become much larger than surface tension force, droplets undergo rolling action on surfaces. As droplet size increases and droplet diameter becomes larger than the capillary length, in general, a combination of droplet sliding and rolling can occur on hydrophobic surfaces [15]. Texture topology of hydrophobic surface also resembles micro/nano porous structures, which are occupied by air on surfaces. Depending on porous sizes, roundness, and pore diameter, droplet motion changes from rolling to sliding. This becomes particularly critical as pore sites are partially filled with liquid such as water condensate in humid air ambient. In such a situation, droplet rolling changes to droplet sliding mode on surfaces [16,17]. Moreover, changing of droplet mode of rolling/sliding becomes possible as wetting state changes reversibly [18]. The initiation of droplet rolling on surfaces is also possible from external radiative heating; hence, the flow created in droplet fluid results in an imbalance of momentum while initiating droplet rolling motion on hydrophobic surfaces [19]. Stretchable hydrophobic surfaces can alter wetted diameter on surfaces and allows the mode of droplet motion. This is mainly because of surface stretching, which increases the wetting diameter of the droplet while enhancing droplet adhesion on surfaces. Hence, increased pinning force retards droplet motion from rolling to sliding [19]. 

The mode of droplet motion on the hydrophobic surface is critical for dust mitigation from surfaces. The mode of sliding causes large droplet puddling and wetted area expansion on surfaces. In such situations, dust mitigated from the hydrophobic surface cannot be carried by a large amount to the droplet inside by flow currents because of small droplet height due to the wetting diameter extension under sliding. In addition, the rolling mode of large size droplets (≥30 µL) suffers from large puddling, which gives rise to striation edge features in the droplet paths on dusty hydrophobic surfaces. This creates geometrically-irregular cleaned surfaces as compared to that created by small volume droplets [20]. Using small volume droplets (≤20 µL) generates a parallel sided narrow droplet path on dusty surfaces; however, the amount of dust picked up by rolling droplets becomes small due to the narrow droplet path. On the other hand, the spinning of rolling droplets can minimize striations, like edges, along the droplet path for large volume droplets (≥30 µL). In the present study, a novel mechanism for the spinning of a rolling droplet is introduced and spinning/rolling droplet dynamics on clean and dusty hydrophobic surfaces are examined. An experiment was carried out to simulate the spinning of rolling droplets and dust mitigation from surfaces. A wedge fixture was designed and realized towards generating droplet spinning during rolling. In the fixture design, V-shaped and inclined hydrophobic surfaces with different wetting characteristics (contact angles and hysteresis) on each surface were considered. Droplet rolling in V-shaped surfaces experiences different pinning on each hydrophobic plate, forming the wedge while creating rotational momentum on a rolling droplet in the wedge. A high speed recording system was accommodated to analyze rolling and spinning droplet kinematic characteristics. A mathematical model was also developed resembling droplet behavior in the wedge adopting the conditions of experiments. Droplet kinematic relations including rolling and spinning velocities, and tilting of droplet vertical axis under spinning were compared with those obtained from experiments. Dust mitigation from inclined hydrophobic flat surfaces by a rolling-only droplet, and a rolling and spinning droplet was compared in the frame of self-cleaning applications.

## 2. Experimental

**Figure 1:**
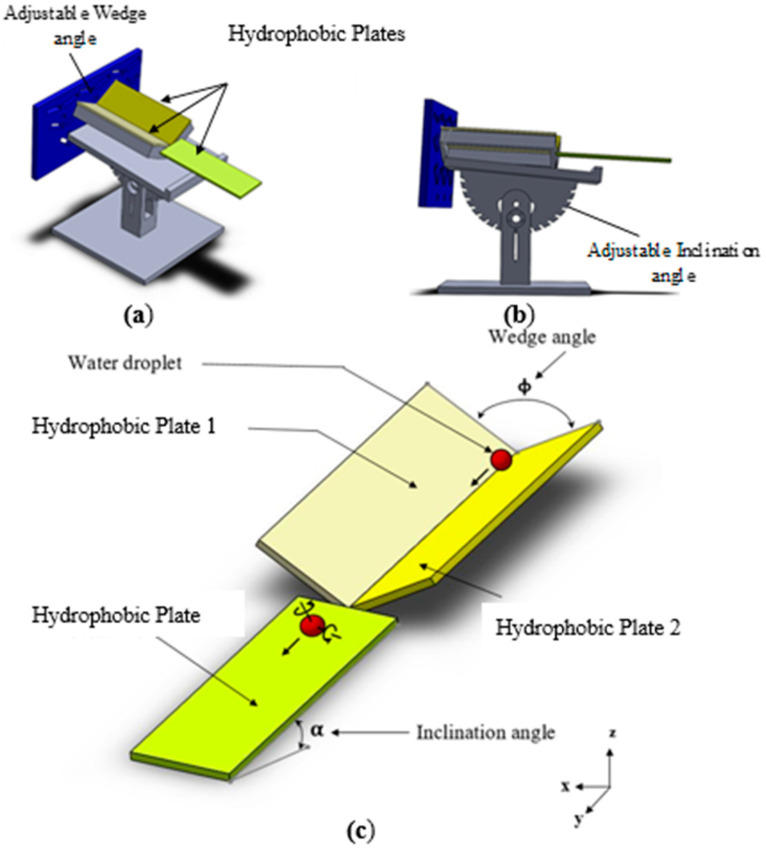
Design and schematic view of inclined wedge fixture: (**a**) Design perspective view of fixture, (**b**) design side view of fixture, and (**c**) schematic view of wedge fixture.

**Figure 2:**
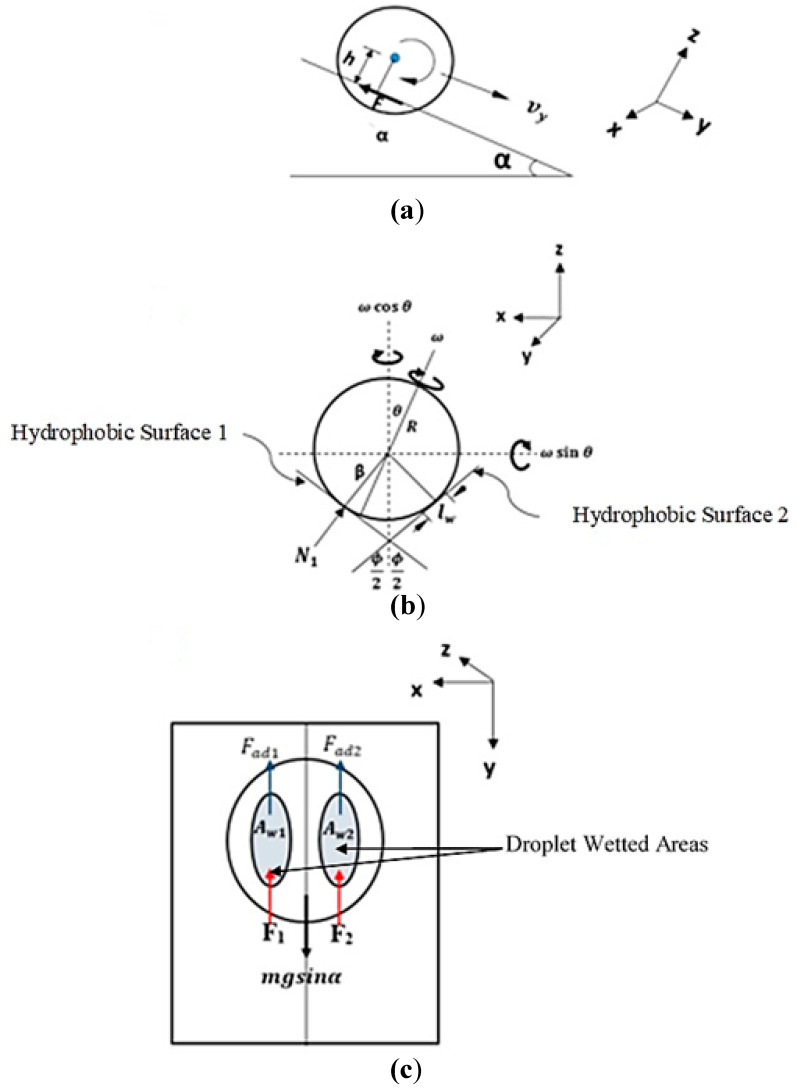
Plane views of droplet in wedge fixture: (**a**) y-z-plane view, (**b**) x-z-plane view, and (**c**) x-y-plane view.

A fixture was designed and manufactured using the MakerRobo UP 3D printer at FABLAB in in King Fahd University of Petroleum and Minerals, Saudi Arabia. The fixture assembly and geometry were designed using SOLIDWORKS 2018. In assembling the wedge fixture, hydrophobic polycarbonate sheets of dimensions 30 mm x 70 mm were used as plane surfaces in the wedge fixture (Figure 1). The fixture enables rolling and spinning water droplets by partially wetting both surfaces of the fixture plates due to the hydrophobic characteristics of plate surfaces. The wetted areas of the droplet are shown schematically in Figure 2. Polycarbonate plate surfaces were hydrophobized (in Laser Laboratory) using two techniques to achieve different hydrophobic states on each polycarbonate plate surface. Hydrophobizing techniques incorporated were: i) Solution crystallization, and ii) functionalized silica particles coating. Solution crystallization involved with immersing of polycarbonate plate into diluted acetone solution (30% acetone by volume) for a short period. It is worth mentioning that in accordance with the early work [21] acetone concentration was kept 30% in the solution and immersion duration of polycarbonate plate was set at 4 min. Immediately after the immersion duration, polycarbonate plates were rinsed with desalinated water and dried in an ambient atmosphere. In the case of silica particle coating of the polycarbonate plate surface, silica particles were synthesized and functionalized before coating surfaces similar to the coating described in the early work [15,22]. During the synthesizing phase, tetraethyl orthosilicate (TEOS), isobutyltrimethoxysilane (OTES), ethanol, and ammonium hydroxide were incorporated and 14.4 mL of ethanol, 1 mL of water, and 20 mL of ammonium hydroxide were mixed for 20 min. The modified silane was supplied in a molar ratio of 3:4 to the mixture and mixed for 12 h in the laboratory environment. Dip coating was introduced for depositing the mixture onto polycarbonate plate surfaces. Coating surfaces were treated via centrifuging and washing by ethanol towards removing reactants from surfaces. 

JEOL 6460 SEM (scanning electron microscope) was employed for evaluating the surface texture of crystallized and coated surfaces. The Park NX10 atomic force microscopy (AFM) was used to assess the crystallized and coated plate surface profiles. Droplet contact angles on crystallized and coated surfaces were evaluated by the goniometer incorporating technique used in the early study [23]. A high-speed recording facility (SpeedSense 9040, Dantec Dynamic (Skovlunde, Denmark)) was used to assess the droplet motion in the wedge fixture. A high speed camera was operated at 5000 frames-per-second (fps) with resolution of 1280 × 800 at 14 µm × 14 µm pixel size. Recording tests were repeated securing repeatability of the data recorded. Errors because of the repeats of data recording was determined to be about 3.5%. Uncertainty (±*u*) for the data recorded was evaluated via using a range of data around the measurement points (droplet locations) and errors related to the displacement of data points (in terms of pixels). As 96% confidence-level was achieved for displacement data basing on repeatability, the standard deviation of data fitting a Gaussian error distribution was obtained and it was about 2%. The equation for estimating standard uncertainty (σ_u_) is [24]:

(1) $$\sigma_{u}=\sqrt{\int_{x_{o}}^{x_{n}}{\left(x-\mu_{e}\right)}^{2}p\left(x\right)\mathrm{dx}}$$

where, µ_e_ being the mean value of x, n represents the number of data points, and p(x) corresponds to probability distribution function. The probability distribution function for all droplet displacements influencing droplet velocity was first found from the instant correlation-plane. The probability distribution function was, later, related to a suitable Gaussian-function through which the probability distribution function diameter was obtained. Standard uncertainty was evaluated adopting a least-squared-Gaussian-fit and the end findings were structured (normalized) by the number of pixels those contributing to cross-correlation-peak. Bias error related to measurement was observed to be about 0.02 pixels, which occurred because of the complexity of the sizing of very small-peaks in the probability distribution function. Consequently, standard uncertainty was determined as about 3.5%.

**Figure 3:**
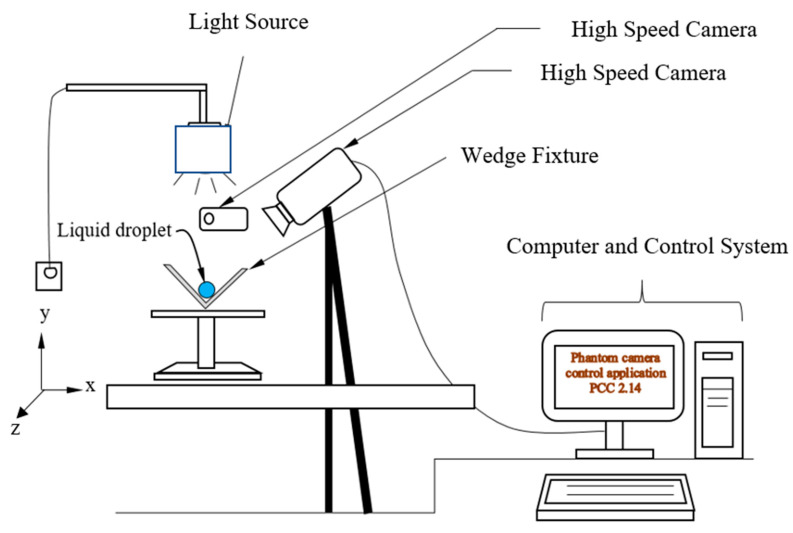
Experimental set-up for a droplet on inclined wedge fixture.

To determine rolling and spinning droplet mitigating dust particles from hydrophobic surfaces, environmental dust was gathered by soft brush from photovoltaic cover glass surfaces in King Fahd University of Petroleum and Minerals Saudi Arabia campus. Collected dust was examined and their sizes, density, shapes, and constituting compositions recorded. The average dust particles were measured by a particle size analyzer and it was about 1.2 µm and averaged dust density was 2800 kg/m^3^. An experimental rig was realized to monitor droplet motion on wedge fixture. Figure 3 shows the experimental arrangement. A computer system was used to control the trigger and capture of the high-speed camera via Phantom (PCC 2.14) video player/recorder, which allowed to operate digital data recorded at the different number of frames per second.

## 3. Results and Discussion

Rolling and spinning liquid droplets on inclined wedge fixture (V-shape) consisting of hydrophobic surfaces with different wetting states were studied towards achieving improved droplet path geometry on dusty surfaces. Rolling and spinning velocities of droplets in the wedge fixture were evaluated using the analytical approach and behavior of droplets was assessed experimentally by high speed recording system. Environmental dust was characterized before droplet rolling and spinning experiments.

### 3.1. Hydrophobizing of Wedge Surfaces and Dust Properties

**Figure 4:**
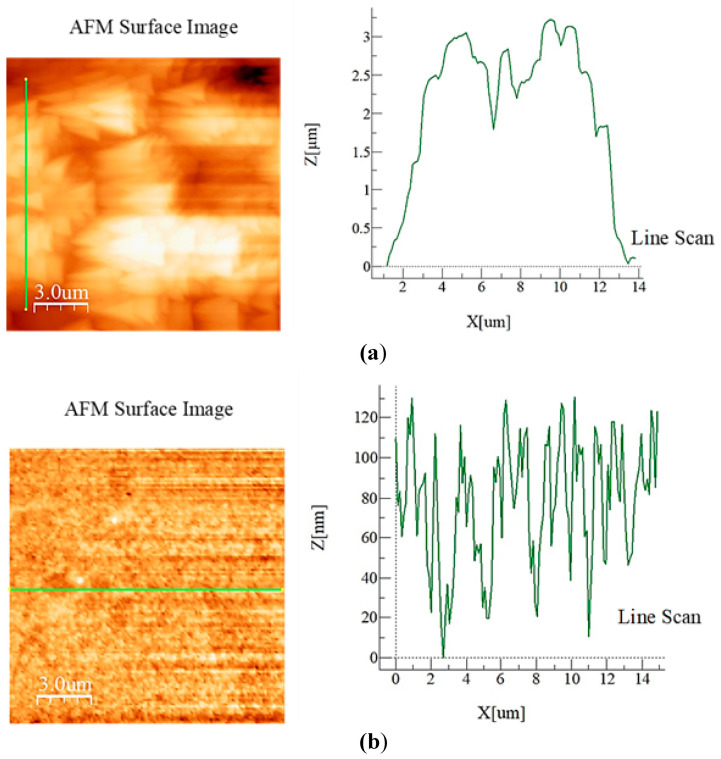
Atomic force microscope images and line scans: (**a**) Crystalized polycarbonate surface, and (**b**) nano-silica coated polycarbonate surface.

Polycarbonate sheets were used to form wedges in the wedge fixture. In order to create different hydrophobic wetting states on each surface of the polycarbonate plates, two different surface treatment processes were adopted. Crystallized plate surface in the wedge fixture generated hydrophobic wetting state with a contact angle of 132° ± 3° and hysteresis of 26° ± 2° while the other surface was coated by functionalized nano-silica creates contact angle of 152° ± 2° and hysteresis of 8° ± 2°. Figure 4a,b depict AFM topology and surface line-scans of crystalized and coated surfaces, respectively. The solution crystallized polycarbonate surface was composed of micro-size semi-spherical structures with randomly oriented on the surface. In addition, nano-fibers emanating from semi-spherical structures were related to secondary crystallization sites on the surface [21]. During acetone treatment, crystals developed at nucleation centers, and they branched radially on polycarbonate surfaces. As time progressed, semi-spherical crystals were formed from the intermittent branch sites [25,26]. Acetone possesses a solubility parameter (Hildebrand) of 20.1–20.3 J^1/2^cm^-3/2^ [27], which demonstrates miscible behavior. Hence, acetone diffusion through a polycarbonate surface develops a soft layer (gelation-like structures) and transition-glass-temperature of polymeric structures next to the vicinity of the soft-layer became less, which resulted in plasticizing of the polymer in this region [27]. The gelatin-like sites (soft film) triggerred crystallization initiation and nucleus sites in the form of the bundle and lamellar features. Repeating accumulation of crystallized units gradually formed semi-spherical structures on the surface. Moreover, acetone diffusion was in the non-Fickian form [28,29]; hence, the diffusion front propagated at an almost constant speed towards polycarbonate micro-structures [29,30]. Hence, keeping polycarbonate in the acetone bath further increased the crystal size and depth of the crystallized layer. However, the immersion duration was kept to 4 min to avoid excessive crystallization on the surface. It is worth mentioning that excessive crystallization on the surface lowered optical transmittance and contact angle hysteresis [21], which enhanced droplet pinning on surfaces. Moreover, small pores and cavity-like structures contributed to the topology of the crystallized surface. Topologically, the surface was made up of micro-pore hill-like structures as depicted from the line scan of the surface (Figure 4a). The average roughness of the crystalized surface was about 2.5 µm. Moreover, for nano-silica unit-coated samples, nano-size spherical silica units (~30 nm) were observed on the coating surface (Figure 4b). Nano-size silica agglomerates formed texture structures, which were composed of nano-size valley and hills. This was apparent from surface line scan as shown in Figure 4b. The average roughness of the coating surface was about 75 nm. Consequently, hydrophobizing of plates with different wetting states was possible in the inclined wedge fixture (Figure 3). The contact angle and hysteresis differences of both plates in the wedge fixture gave rise to different stage of pinning of droplet on surfaces of plates.

### 3.2. Droplet Rolling and Spinning Dynamics

Adhesion of rolling droplet on hydrophobic surface was governed by droplet receding and advancing angles, wetted diameter, surface tension, and roughness parameter of surface. Roughness parameter was defined through a ratio, which was the same as area of hills covered on the surface divided by total projected area of the surface. The crystallized surface has a roughness parameter of 0.4 while the coated surface has 0.6. The pinning force of rolling droplet is $F_{pin}=\frac{24}{\pi^{3}}\gamma fD_{w}\left(cos\theta_{R}-cos\theta_{A}\right)$ [31], here, γ is surface tension, f is roughness parameter, D_w_ is wetting diameter, and θ_R_ and θ_A_ are receding and advancing angles of rolling droplet. Hence, each plate in the wedge fixture created different pinning forces on rolling droplets while generating rotational moment through which the spinning motion resulted on rolling droplets, i.e., droplet spins while rolling down the inclined wedge fixture. It is worth mentioning that care was taken to avoid excessive adhesion of droplet fluid on plate surfaces, which can cause breaking up of rolling and spinning droplets because some droplet fluid can adhere plate surfaces.

**Figure 5:**
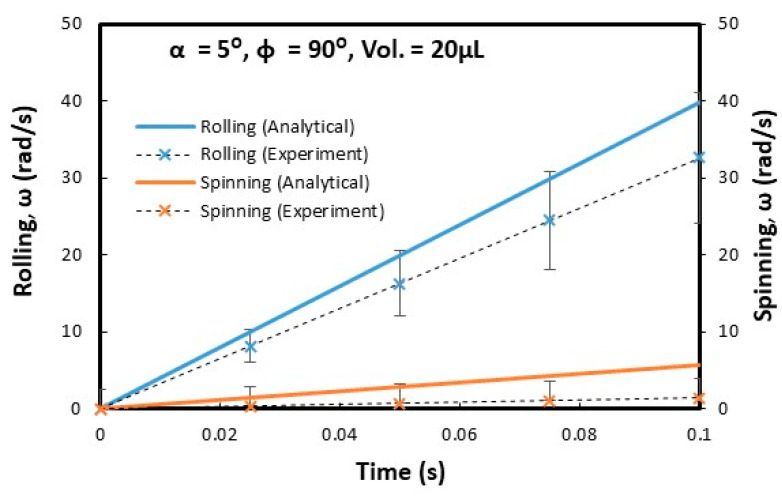
Rolling and spinning velocities of the droplet with time in wedge fixture obtained from experiments and predictions obtained using Equations (5) and (6). α and  ϕ are the inclination and wedge angles.

**Figure 6:**
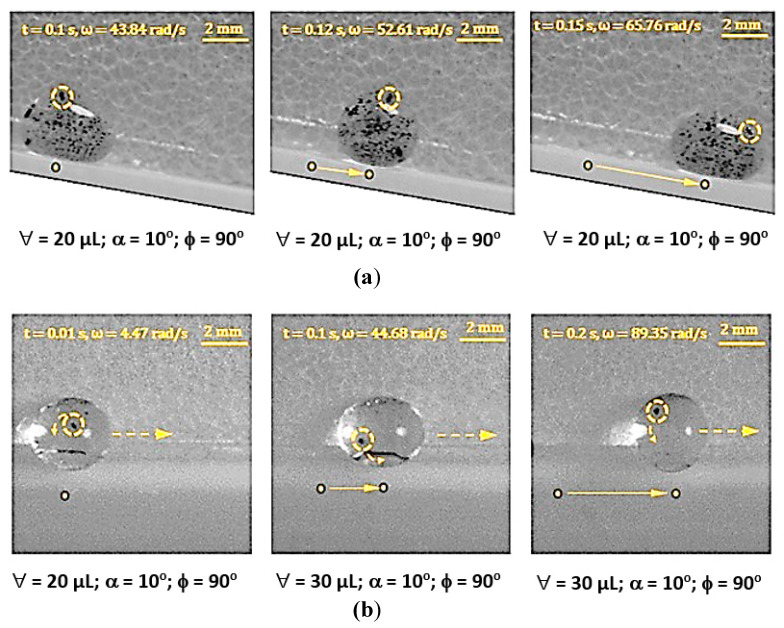
High speed camera images of rolling and spinning droplet: (**a**) Rolling droplet images, and (**b**) spinning droplet images. High speed recorded data are analyzed by the tracker program to evaluate rolling and spinning droplet velocities. The darker color in droplet fluid represents clustered carbon nanotubes. α and  ϕ are the inclination and wedge angles.

**Figure 7:**
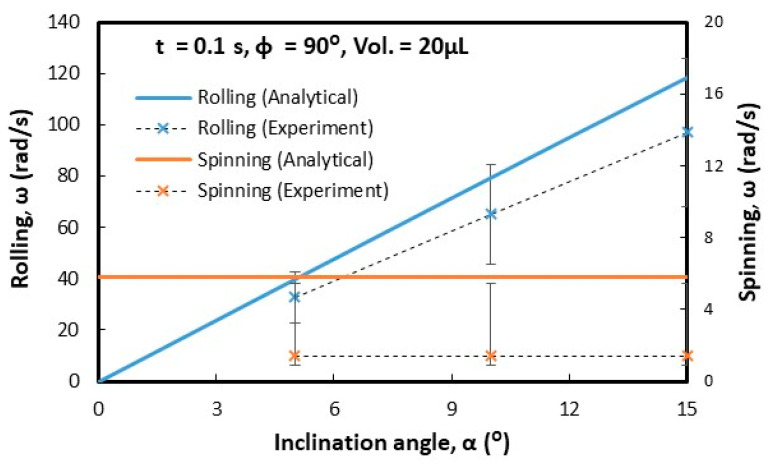
Rolling and spinning velocities of the droplet with fixture inclination angle (α) obtained from experiments and predictions obtained using Equations (5) and (6). α and  ϕ are the inclination and wedge angles.

**Figure 8:**
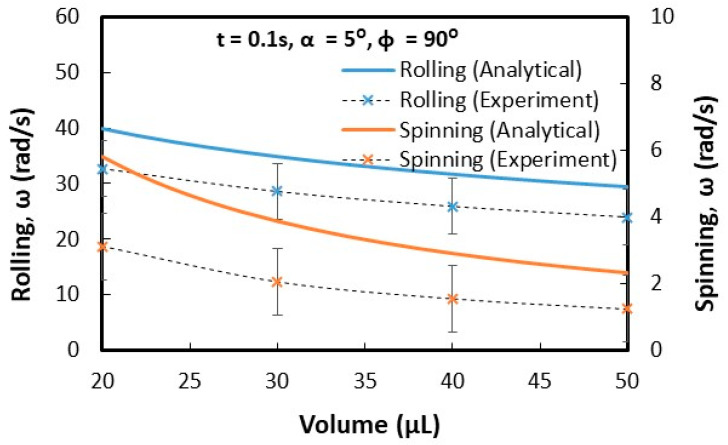
Rolling and spinning velocities of the droplet with droplet volume (∀) in inclined wedge fixture obtained from experiments and predictions. α and  ϕ are the inclination and wedge angles.

**Figure 9:**
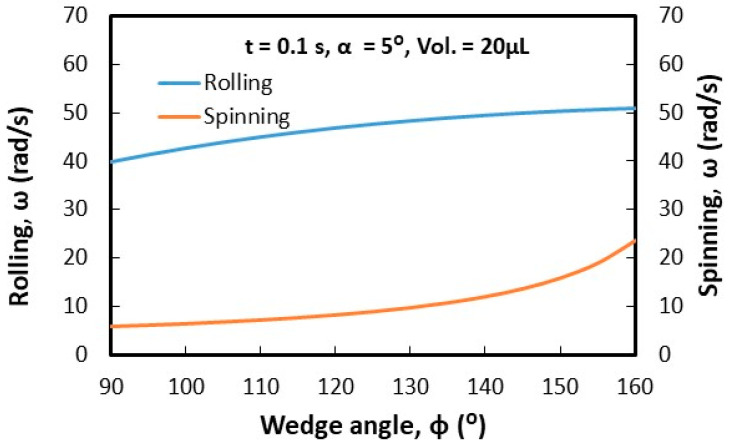
Predicted rolling and spinning velocities of the droplet with wedge angle (ϕ) in wedge fixture. α and  ϕ are the inclination and wedge angles.

Droplets under gravity puddle and change shape from spherical to spherical-ellipsoid; the puddle thickness (height) depends on surface tension, fluid specific gravity, and surface contact angle via relation through $\sqrt{2\left(1-\cos\theta \right)\frac{\gamma }{\rho g}}$, here, θ is contact angle, γ being surface tension, ρ is the fluid density, and g corresponds to gravity [32]. Droplet puddle alters the center of droplet mass during rolling because of droplet wobbling on the surface. However, for small size droplets, which is comparable to capillary length ($\kappa^{-1}=\sqrt{\frac{\sigma }{\rho g}}$, here $\kappa^{-1}$ is the capillarity length), the droplet rolls like a spherical marble without undergoing wobbling. For large droplet volumes, the droplet puddles and undergoes elastic deformation and advancing (θ_A_) and receding (θ_R_) angles change dynamically during its transition on the surface. Although the equation of motion for rolling droplet is described previously [4], it takes a different form for rolling and spinning droplets in the inclined wedge shape fixture. The rotational motion of droplet during rolling takes the form (Equation(1)) $\frac{d\omega }{\mathrm{dt}}=\frac{\mathrm{At}}{\omega }+K\sqrt{\left(1-\frac{{\mathrm{At}}^{2}}{\omega^{2}}\right)}$, where A=a2R2sin2(ϕ2) and $K=\frac{120\gamma \left[f_{1}\left(\cos\theta_{R1}-\cos\theta_{A1})-f_{2}(\cos\theta_{R2}-\cos\theta_{A2}\right)\right]}{{\rho \pi }^{3}\forall \cos\left(\frac{\phi }{2}\right)}$. Here, ω is the angular velocity of rolling droplet, a=gsinα1+25sin2(ϕ2), R is droplet radius when it is spherical, ϕ is wedge angle between the plates in the fixture (V-shape), α is the inclination angle of the wedge fixture, $\theta_{R1}$ and $\theta_{R2}$ are receding angles of droplet on plates 1 and 2 in the wedge fixture, $\theta_{A1}$ and $\theta_{A2}$ are advancing angles of droplet on plates 1 and 2 in the wedge fixture, and ∀ is droplet volume. The time corresponding to change of rotational velocity ($\frac{d\omega }{\mathrm{dt}}$) remains at zero as droplet rotational velocity (ω) becomes constant during rolling. This condition requires that $=\sqrt{\frac{K}{A^{2}t^{2}\left(1+\frac{K}{A}\right)}}$; hence, for a fixed wedge geometry (V-shape) and droplet size, A remains constant, while K changes because of variation of $\theta_{R1}$, $\theta_{R2}$, $\theta_{A1}$, and $\theta_{A2}$ during droplet wobbling under puddling. Therefore, for small size droplet, which can be similar to capillary length size ($\kappa^{-1}=\sqrt{\frac{\sigma }{\rho g}}$), constant rotational velocity of rolling droplet can possibly be achieved in the wedge fixture. Alternatively, for large droplets constant rotational velocity cannot be maintained along with the inclined wedge structure. Droplet spinning is resulted because of pinning forces difference over the contact area of each plate in the V-shaped wedge fixture, i.e., different hysteresis on each plate creates different pinning forces on rolling droplet in V-shaped wedge fixture. During droplet spinning, the spinning axis of droplet changes (θ) because of the angular momentum variation during rolling and spinning. The angle of the spinning axis (θs) is cosθ=(1−(gtsinα1+25sin2(ϕ2))2R2ω2sin2(ϕ2)). As the inclination angle (α) of the wedge fixture reduces and/or rotational velocity (ω) of droplet increases cosθ approaches to unity and spinning occurs normal to the rolling direction. Figure 5 shows the time variation of rotational velocity that resulted from analysis and was obtained from experiments for 20 µL droplet at ϕ = 90° wedge angle and α = 5° inclination angle of the wedge fixture. In addition, experimental values are also shown for comparison. The angular velocity of droplet rotation and spinning increases with time. The angular velocity of rolling becomes larger than the angular velocity of spinning. Hence, gravitational influence becomes more influential than droplet adhesion on wedge plate surfaces. Experimental data for the angular velocity of rolling and spinning show similar trends of predictions and they have almost close values. The error related to the experiment is about 3.5%, which is based on the repeatability of droplet rolling and spinning tests. In addition, the assumption of the constant droplet mass center location through rolling and spinning, in the analysis, contribute to differences between experimental findings and predictions. Nevertheless, they have the same trend with close values. Figure 6 shows rolling and spinning droplet images at different locations in the inclined wedge fixture. To evaluate droplet rolling and spinning velocities, carbon nanotube clusters (5 µm) were inserted in droplet fluid by 0.1% volume ratio. Hence, the tracker program was used to assess rolling and spinning velocities from the high speed recorded data. Although droplet size was small (20 µL), light droplet bulging because of puddling was noticed. However, no break up of droplet fluid on wedge plate surfaces was observed from the droplet images. The influence of the inclination of the wedge fixture angle (α) on droplet rolling and spinning was also examined, which is shown in Figure 7. Increasing the wedge fixture inclination angle gave rise to increased droplet rolling velocity; however, spinning velocity remained almost the same for all inclination angles. This is related to the uniform wetting state of wedge plates in the fixture, which generates uniform droplet pinning along plate surfaces during rolling. However, experimental values remained lower than those of the predictions. Nevertheless, they showed the same trend with an increasing wedge fixture inclination angle. The influence of droplet volume (∀) on rolling and spinning velocities is shown in Figure 8. Experimental data is also shown for comparison. Increasing droplet volume reduced both rolling and spinning velocities despite the weight of droplet increasing with droplet size. This was due to the increased wetted area on wedge plate surfaces for large size droplets while altering advancing and receding angles. Hence, the droplet pinning force on plate surfaces became different as the droplet volume increased. Consequently, this influenced droplet rolling and spinning in the wedge fixture towards a reduction in both velocities. Experimental findings showed a similar trend as of those of predictions with increasing droplet volume. The effect of wedge angle (ϕ) of the fixture on rolling and spinning velocities are shown in Figure 9. Increasing the wedge angle enhanced droplet rolling and spinning velocities. Hence, increasing the wedge angle lowered the wetted diameter of the droplet on wedge plates, which was because of droplet weight, i.e., for small wedge angles, the droplets bulged in between plates in the wedge fixture while increasing the wetted area on the plate surface. Pinning forces on plate surfaces decreased with reducing droplet wetted diameter on plate surfaces, which enhancing droplet rolling velocity. Alternatively, the difference between forces of pinning on plate surfaces became large as the wedge angle increased. This increased the droplet angular momentum in the spinning direction, i.e., spinning velocity increased. In addition, increased spinning causes a shift of droplet vertical axis towards the spinning direction. Hence, the spinning angle became further off from the droplet vertical axis, i.e., it increased from 1.45° to 1.53° as the wedge angle increased from 90° to 150° since a fat inclined hydrophobic plate was placed in front of the wedge fixture.

**Figure 10:**
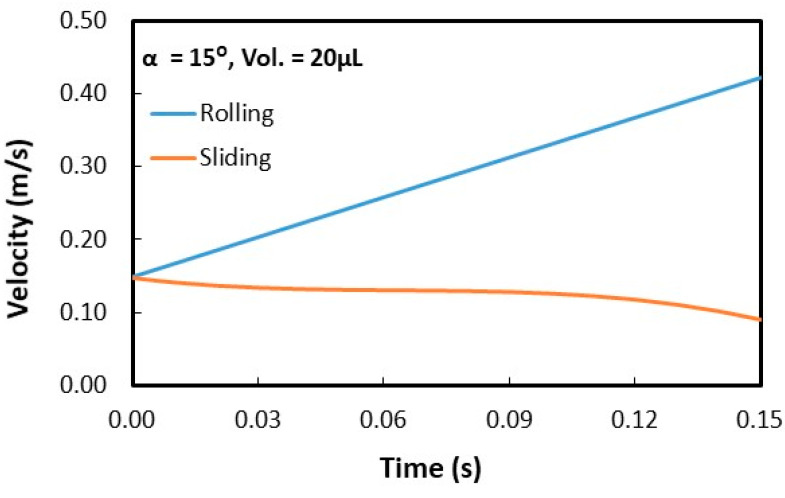
Predicted rolling and spinning velocities of the droplet with time on the inclined plane hydrophobic surface. α and  ϕ are the inclination and wedge angles.

Droplet behavior was analyzed by incorporating the analysis presented in the previous study [4]. Figure 10 shows rolling and spinning velocities of droplet for 15° angles of the flat hydrophobic plate. Droplet rolling velocity increased while spinning velocity reduces on the surface with increasing time. Hence, it reduced droplet spinning velocity by almost 35% of the initial spinning velocity. Hence, as droplet moved onto the inclined flat hydrophobic plate from the wedge fixture, droplet initial velocity (upon touching on the flat hydrophobic surface from the wedge fixture) increases considerably and droplet spinning reduces on the flat hydrophobic surface. It is worth indicating that droplet motion on a flat hydrophobic surface is initiated upon leaving the wedge fixture. The contact angle of the inclined flat hydrophobic surface is 152° ± 2° with a hysteresis of 8° ± 2°. Moreover, the Bond number for rolling droplet is: $\frac{{\rho \omega }^{2}R^{3}}{8\gamma }$, where ρ is density, R being droplet radius, ω being droplet rotational velocity and γ is surface tension. As droplet angular rotation (ω) and droplet size becomes large, the Bond number attains large values. However, the Bond number is the ratio of centripetal force over surface tension force for rolling droplets. Hence, for large Bond numbers, centripetal force becomes large, which causes almost constant location of droplet mass center during rolling. This reduces wobbling of rolling droplets on surfaces [4]. In addition, the ratio of rolling velocity (tangential) over droplet linear velocity (ωR/V) influences droplet wobbling due to the change of pressure differential between droplet liquid and its ambient [33]. However, dynamic change of pressure differential becomes critical for wobbling and it is evaluated by $\varphi =\frac{\Delta {\rho \omega }^{2}R^{2}}{\rho_{a}V^{2}}\gg 1$, here Δρ being density variation of droplet fluid and air surroundings and ρ_a_ is density of air. For droplet sizes considered 20–40 µL, value of φ changes between 800 to 950, i.e., $\frac{\Delta {\rho \omega }^{2}R^{2}}{\rho_{a}V^{2}}\gg 1$. This shows that dynamic pressure differential has not significant influence on wobbling as indicated in the early work [33]. 

**Figure 11:**
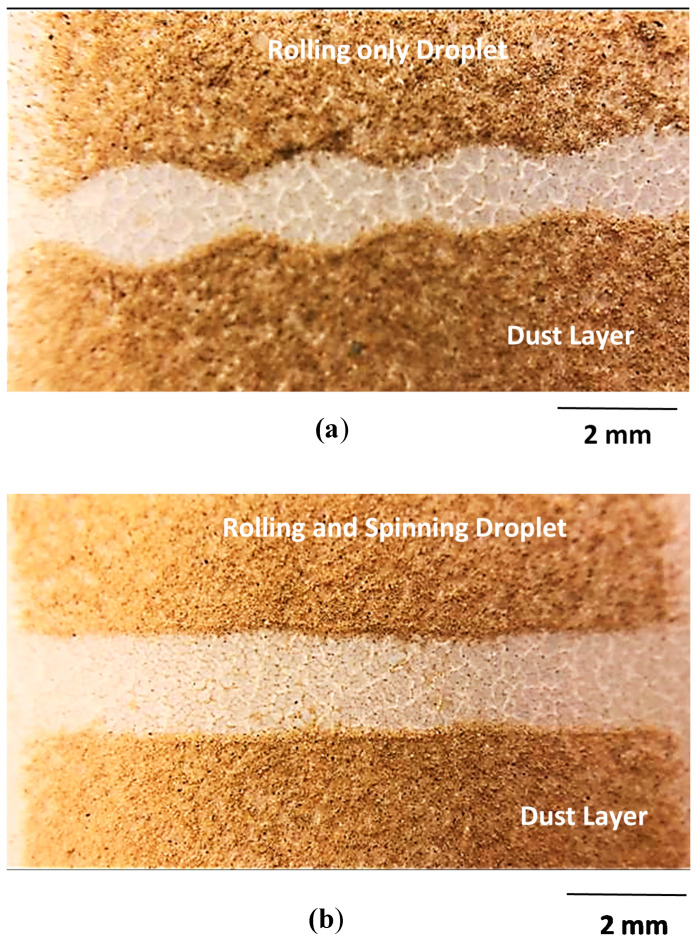
Optical image droplet path on the dusty hydrophobic surface: (**a**) Rolling only droplet path, and (**b**) rolling and spinning droplet path.

Figure 11 shows an optical image of droplet paths on dusty hydrophobic surfaces. Rolling and spinning and rolling only (non-spinning) droplets with the same volume (20 µL) were used in the experiments to obtain corresponding droplet paths. Striation-like structures at the edges of the droplet path was observable for rolling only (non-spinning) droplet. This is because of droplet bulging during rolling under puddling influence. It is worth noting that in the puddling motion of the droplet, the droplet mass center reduced by λ as a reference to the rolling surface. Differences in energy potential between spherical and puddled droplets were approximately: γ λ^2^ ≅ ρgR^3^, here R being the droplet radius [34]. Wetted length of a puddled droplet on the surface can be approximated as:$\;l=\sqrt{R\lambda }$. Potential energy minimization of droplets having same radius, but different shapes (puddle and round) in relation to wetted length results in:${\;\rho \mathrm{gR}}^{3}\lambda \sim {\gamma l}^{4}/R^{2}$. Therefore, wetted length of the droplet puddled yields: $l≅R^{2}/\sqrt{\frac{\gamma }{\rho g}}$. However, similar expression is also provided in the early work [34]. The term $\sqrt{\frac{\gamma }{\rho g}}$ is droplet capillary length. This yields potential energy minimization in the form of: ${\rho \mathrm{gR}}^{3}\lambda \sim \gamma_{f}l^{4}/R^{2}$. Hence, shift in droplet center of mass (λ) yields: ~ R^3^$/\frac{\gamma }{\rho g}$. Padding of the droplet alters the maximum droplet height, i.e., for 20 µL, λ yields about 0.50 mm, which is small. However, for large droplets (>30 µL), puddling cannot be avoided and λ takes values comparable to 1 mm and more. Moreover, in the case of a spinning droplet, edge defects (striation-like edges) disappear and parallel sided edges result along the droplet path. Hence, a rolling and spinning droplet removes dust along its path with parallel edges during its transition on dusty surface, i.e., spinning contributes to dust removal from path edges. Dust is mitigated by droplet fluid over droplet wetted area on dusty surface [15]. To evaluate the area clean along the droplet path, a cleaning factor is introduced, which corresponds to the area of dust removed by the droplet over a rectangular area of the surface as if the droplet perfectly cleans the surface (highly parallel sided cleaning along droplet path). The cleaning factor remains higher for a rolling and spinning droplet than a rolling only droplet on an inclined plane dusty surface. Hence, spinning adds to the cleaning factor on dusty hydrophobic surfaces.

## 4. Analytical Modeling of Rolling and Spinning Droplet on Inclined Wedge Fixture

Figure 1 shows a schematic view of the inclined wedge fixture having two surfaces of different hydrophobic properties. The liquid droplet can rotate and spin while transiting along the wedge length. Forces acting on liquid droplets are shown schematically in 2-dimensional frames. The liquid droplet is assumed to maintain a spherical shape having two wetted areas being in contact with hydrophobic wedge plate (wall) surfaces. Maintaining the spherical shape of the droplet in the wedge fixture is possible as droplet size remains small, i.e., small droplets remain spherical on hydrophobic surfaces despite the wetted area is created on the surface [32].

Formulation of droplet motion in wedge fixture is provided in the Appendix A and only the final equations are provided herein. Considering the rate of momentum change in x, y, and z-axes in relation to droplet motion in the inclined wedge fixture (Figure 2), the following equations have resulted for droplet rolling and spinning motions:

(2) $$\frac{d\omega }{\mathrm{dt}}=\frac{\mathrm{At}}{\omega }+K\sqrt{\left(1-\frac{{\mathrm{At}}^{2}}{\omega^{2}}\right)}$$

where $K=\left[-\omega \;\sin\theta \frac{d\theta }{\mathrm{dt}}+\cos\theta \frac{d\omega }{\mathrm{dt}}\right]$, A=a2R2sin2(ϕ2)~ s−4, and a is the linear acceleration along the y-axis (a =dvydt=gsinα1+25sin2(ϕ2)), ω is the droplet rotational velocity, θ is the angle of the spinning axis, t is time, R is droplet radius, ϕ is the wedge fixture angle (V-shape angle), and α is the wedge fixture inclination angle (Figure 2), g is gravity. 

The resulting equation (Equation (1)) for angular acceleration of rolling and spinning droplet is a nonlinear ordinary differential equation of first order and it depends on angle of spinning axis, wedge fixture angle, wedge inclination angle, wetting states of hydrophobic plate surfaces, droplet fluid properties, and size of the droplet. The solution for the non-linear ordinary differential equation (Equation (1)) is obtained from Wolfram Alpha software (Mathematica Program). The solution yields:

(3) $$\omega \left(t\right)=\sqrt{t^{2}\left(A+K^{2}\right)+2{\mathrm{Kte}}^{c1}+e^{2c1}}$$

Imposing the initial condition as initially no rolling and spinning of the droplet (ω(0)=0) in Equation (2), a specific solution can be obtained as:

(4) $$\omega =\;t\sqrt{\left(A+K^{2}\right)}$$

Inclination of spinning axis of droplet (θ) yields:

(5) θ=sin−1(gt sinαRt(A+K2)sinϕ2(1+25sin2(ϕ2)).)

Droplet rolling (or rotation) angular velocity, $\omega_{x}\left(t,\;A,\;K,\;\alpha ,\;\phi ,\;R\right)$ can be obtained as:

(6) ωx=gt2 sinαRsinϕ2(1+25sin2(ϕ2)).

Droplet rolling angular velocity ($\omega_{x}$) depends on: time, inclination angle, wedge angle, and droplet size.

Droplet spinning angular velocity, $\omega_{z}\left(A,K,\;\phi ,\;R\right)$ yields:

(7) ωz=(t2(A+K2)−(1+25sin2(ϕ2))2R2sin2(ϕ2).)

Unlike rolling speed, droplet spinning speed ($\omega_{z}$) is function of fluid properties, droplet size, hydrophobic surface wetting state, and wedge angle. Equations (5) and (6) are used for computing angular rotational and spinning velocities of droplets in the inclined wedge fixture. Table 1 gives the properties of hydrophobic surfaces in the wedge fixture and water used in the computations.

## 5. Conclusions

To achieve a parallel-sided droplet path on dusty hydrophobic surfaces, droplet spinning was introduced prior to droplet motion on dust surfaces. The wedge fixture was designed and built. The wedge fixture walls were modified to place hydrophobic surfaces with different contact angle hysteresis. This provided the spinning and rolling of water droplets during their transition along the wedge fixture. Contact angles and hysteresis of each hydrophobic plate (wedge wall) in the wedge fixture were 132° ± 3° with a hysteresis of 26° ± 2° for crystallized surface while it is 152° ± 2° with a hysteresis of 8° ± 2° for functionalized nano-silica coated surface. An inclined hydrophobic flat plate was located at the wedge fixture end. This arrangement enabled the movement of a rolling and spinning droplet smoothly transiting onto the inclined flat hydrophobic surface. Hence, droplet rolling and spinning initiated in the wedge fixture could continue rolling and spinning during droplet transiting on an inclined hydrophobic flat surface. Droplet motion in hydrophobic wedge fixture assembly and flat surface was formulated and findings were compared with experiments by using a high-speed droplet motion recording system. Experiments were repeated for dust mitigation by rolling and spinning and rolling only droplets from flat dusty hydrophobic surfaces. The area cleaned on inclined flat dust surface by rolling and spinning droplet was compared with that of one cleaned by rolling droplets only. Findings revealed that introducing the inclined wedge fixture with different hydrophobic wall characteristics created droplet rolling and spinning along the length of the fixture. The angular velocity of droplet rolling was much larger than the angular velocity of droplet spinning in the wedge fixture. The increased inclination angle of the fixture enhanced droplet rolling velocity as compared to droplet spinning velocity. This is because of the influence of gravity on droplet motion. Since wetting states of hydrophobic plates in the fixture remained almost uniform along the wedge length, the radial momentum remained almost constant (due to constant droplet pinning force on plate surfaces) during droplet transition along the wedge fixture length. This resulted in an almost constant spinning velocity of the rolling droplet. Increasing droplet volume lowered both rolling and spinning velocities along the wedge fixture length because of increased wetted length on the wedge plate surfaces. Droplet spinning velocity reduced on the inclined flat hydrophobic surface while droplet rolling speed increased. Rolling and spinning droplets on dusty flat hydrophobic surface resulted in parallel-sided droplet path unlike the rolling droplet only case, which gave rise to striation-like edges on the droplet path because of the droplet wobbling under puddling influence. The area cleaned by a rolling and spinning droplet on the dusty inclined flat hydrophobic surface was larger than its counterpart corresponding to droplet rolling only case.

## Figures and Tables

**Table 1:** Properties of distilled water and hydrophobic plate surfaces.

S/N	Property	Name	Value	Unit
1	ρ	Density	997.1	Kg/m3
2	γ	Surfaces tension	7.2 × 10^−2^	N/m
3	$f_{1}$	Roughness parameter	0.6	-
4	$\theta_{A1}$	Advancing angle, surface 1	158	Degree
5	$\theta_{R1}$	Receding angle, surface 1	132	Degree
6	$f_{2}$	Roughness parameter	0.4	-
7	$\theta_{A2}$	Advancing angle, surface 2	161	Degree
8	$\theta_{R2}$	Receding angle, surface 2	159	Degree

## Appendix A. Analysis of Droplet Motion in Inclined Wedge with Hydrophobic Flat Surfaces

Liquid droplet in the inclined hydrophobic wedge fixture demonstrates different dynamic behavior than that of the flat inclined hydrophobic plane. Hence, the analysis of droplet dynamics based on force and energy balance is presented for inclined wedge fixture having wedge flat plates with different hydrophobic states. Figure 1 shows a water droplet in the wedge fixture while Figure 2 shows two-dimensional schematic views of the droplet in the wedge fixture. In the analysis, the liquid droplet is considered to be spherical having contacted (wetted) areas on hydrophobic wedge plates located in the fixture (Figure 2). It is worth to note that droplet size comparable to capillary length rolls like a spherical marble on surfaces; however, as droplet volume exceeds 30 µL, droplet wobbling results on hydrophobic surfaces [15]. In the analysis droplet volume considered to be 20 µL and stays assumed to be spherical in the wedge fixture having contact surfaces on the wedge plates.

Force balance in the x-axis (Figure 2) constitutes that:

(A1) $$\sum F_{x}=0$$

and

(A2) $$N_{1}\cos\alpha \sin\beta -{\;N}_{2}\cos\alpha \sin\beta =0$$

where N1 and N2 are normal forces acting on hydrophobic plate surfaces in the wedge fixture.

Summation of forces along the y-axis, i.e., in the direction of down inclined wedge plane, can yield acceleration of droplet, i.e.,:

(A3) $$\sum F_{y}=m\frac{{\mathrm{dv}}_{y}}{\mathrm{dt}}\;$$

and

(A4) $$\mathrm{mg}\sin\alpha -2F-{\;F}_{\mathrm{ad}1}-{\;F}_{\mathrm{ad}2}=m\frac{{\mathrm{dv}}_{y}}{\mathrm{dt}}\;$$

here, α is the inclination angle of the wedge fixture, mg is droplet weight, F is the frictional force, and $F_{\mathrm{adi}}$ is adhesion forces due to droplet pinning. The friction coefficient for two plate surfaces is considered to have the same order of magnitude (i.e.,${\;\mu }_{1}\sim \mu_{2}\sim \mu$) as determined experimentally [32]; hence, same normal reaction forces act on two plate surfaces (i.e., $N_{1}=N_{2\;}=N$)., i.e.,: $F_{1}=F_{2}=\mu N$.

After considering summation of forces along the z-axis, it yields: $\sum F_{z}=0$ → or → 2Ncosβ−mgcosα =0. Hence, it yields:

(A5) $$N=\frac{\mathrm{mg}\cos\alpha }{2\cos\beta }$$

It is worth noting that, change in the droplet center of mass is considered to be negligibly smaller as compared to droplet height on the hydrophobic surface. Therefore, the wetting area of droplet remains relatively constant on wedge plate surfaces. The droplet is assumed to be spherical with wetted surfaces on wedge plates during its motion down the inclined wedge fixture as consistent to that presented in the early study [35].

Considering summation of angular momentum of droplet along x-axis, it yields:

(A6) $$\sum \tau_{x}={I\alpha }_{x}$$

where I is the moment of inertia about x-axis and $\alpha_{x}$ is rotational angular acceleration, which takes the form: $h\left(2F+{\;F}_{\mathrm{ad}1}+{\;F}_{\mathrm{ad}1}\right)=\int_{0}^{h+R}\frac{1}{2}{\mathrm{mr}}^{2}\mathrm{dr}*\frac{1}{h}\frac{{\mathrm{dv}}_{y}}{\mathrm{dt}}$, here, h is the height of the center of mass from the hydrophobic surface. Since spherical droplet is assumed, the moment of inertia becomes: $\int_{0}^{h+R}\frac{1}{2}{\mathrm{mr}}^{2}\mathrm{dr}\approx \;\frac{2}{5}{\mathrm{mR}}^{2}$. Hence, it yields:

(A7) $$h\left(2F+{\;F}_{\mathrm{ad}1}+{\;F}_{\mathrm{ad}1}\right)=\frac{2}{5}m\frac{R^{2}}{h}\frac{{\mathrm{dv}}_{y}}{\mathrm{dt}}$$

or

(A8) $$2F+{\;F}_{\mathrm{ad}1}+{\;F}_{\mathrm{ad}1}=\frac{2}{5}m\frac{R^{2}}{h^{2}}\frac{{\mathrm{dv}}_{y}}{\mathrm{dt}}$$

Inserting adhesion and friction forces into Equation (A8), acceleration term becomes:

(A9) $$\frac{{\mathrm{dv}}_{y}}{\mathrm{dt}}=\frac{\mathrm{mg}\sin\alpha }{m+2{\mathrm{mR}}^{2}/5h^{2}}$$

From Figure 2b, h can be expressed as:

(A10) $$h=R\sin\frac{\phi }{2}$$

Hence, Equation (A9) yields:

(A11) dvydt=gsinα1+25sin2(ϕ2)

Since α and ϕ are time-independent variables, integrating Equation (A11) results in:

(A12) vy=gtsinα1+25sin2(ϕ2)

where α is the inclination angle of wedge fixture along the horizontal plane, ϕ is wedge angle formed by two hydrophobic plates and $v_{y}$ is the linear velocity of droplet down inclined the wedge fixture. Hence, Equation (A11) shows that translational acceleration of droplet down inclined wedge fixture depends only on wedge and inclination angles.

After considering the angular momentum of droplet along the z-axis (i.e., along droplet width), the spinning motion of liquid droplet in the wedge fixture is caused by the difference in adhesion forces at contacted areas on hydrophobic plates. Hence, it can be formulated through:

(A13) $$\sum \tau_{z}={I\alpha }_{z}$$

here I is the moment of inertia about the z-axis, and $\alpha_{z}$ is spinning angular acceleration. Furthermore, inserting forces of adhesion, it yields:

(A14) $$F_{\mathrm{ad}1}R\sin\left(\beta \right)-F_{\mathrm{ad}2}R\sin\left(\beta \right)=I\frac{{d\omega }_{z}}{\mathrm{dt}}.$$

From Figure 2b, β can be expressed as:

(A15) $$\beta =90-\frac{\phi }{2}.$$

Rolling and spinning angular velocities, as shown in Figure 2b, can be written as:

(A16) $$\omega_{x}=\omega \sin{\theta \;\mathrm{and}\;\omega }_{z}=\omega \cos\theta .$$

Therefore, Equation (A14) yields:

(A17) $$F_{\mathrm{ad}1}R\cos\left(\frac{\phi }{2}\right)-F_{\mathrm{ad}2}R\cos\left(\frac{\phi }{2}\right)=I\frac{d[\omega \cos\theta ]}{\mathrm{dt}}$$

or

(A18) $$F_{\mathrm{ad}1}R\cos\left(\frac{\phi }{2}\right)-F_{\mathrm{ad}2}R\cos\left(\frac{\phi }{2}\right)=I\left[-\omega \;\sin\theta \frac{d\theta }{\mathrm{dt}}+\cos\theta \frac{d\omega }{\mathrm{dt}}\right].$$

After the rearrangement, it yields:

(A19) $$\frac{\left(F_{\mathrm{ad}1}-F_{\mathrm{ad}2}\right)R\cos\frac{\phi }{2}}{\frac{2}{5}{\mathrm{mR}}^{2}}=\left[-\omega \;\sin\theta \frac{d\theta }{\mathrm{dt}}+\cos\theta \frac{d\omega }{\mathrm{dt}}\right].$$

Since the parameters in the left-hand side (LHS) of Equation (A19) remains constant with time, one can denote LHS of Equation (A19) as K, where $K=\left[-\omega \;\sin\theta \frac{d\theta }{\mathrm{dt}}+\cos\theta \frac{d\omega }{\mathrm{dt}}\right]$. The constant K can be determined from properties of droplet fluid and wetting states of two hydrophobic surfaces (forming the wedge fixture).

The transitional velocity of droplet down inclined wedge fixture (i.e., along the y-axis) can also be obtained from:

(A20) $$v_{y}=\omega_{x}h=h\omega \sin\theta =R\omega \sin\theta \sin\frac{\phi }{2}$$

Hence, the derivative of $v_{y}$ in Equation (A20) yields:

(A21) $$\frac{{\mathrm{dv}}_{y}}{\mathrm{dt}}=R\sin\frac{\phi }{2}\left(\sin\theta \frac{d\omega }{\mathrm{dt}}+\omega \cos\theta \frac{d\theta }{\mathrm{dt}}\right)$$

Combining of Equations (A20) and (A21) via multiplying becomes:

(A22) $$\frac{v_{y}{\mathrm{dv}}_{y}}{\mathrm{dt}}=R^{2}\omega \sin^{2}\frac{\phi }{2}\left(\sin^{2}\theta \frac{d\omega }{\mathrm{dt}}+\;\omega \sin\theta \cos\theta \frac{d\theta }{\mathrm{dt}}\right)$$

Using the definition of the constant K, it can be eliminated in $\frac{d\theta }{\mathrm{dt}}$ in Equation (A22), i.e:

(A23) $$\frac{v_{y}{\mathrm{dv}}_{y}}{\mathrm{dt}}=R^{2}\omega \sin^{2}\frac{\phi }{2}\sin^{2}\theta \frac{d\omega }{\mathrm{dt}}+{\;R}^{2}\omega \sin^{2}\frac{\phi }{2}\cos\theta \left[\cos\theta \frac{d\omega }{\mathrm{dt}}-K\right]$$

or

(A24) $$\frac{v_{y}{\mathrm{dv}}_{y}}{\mathrm{dt}}=R^{2}\omega \sin^{2}\frac{\phi }{2}\left[\frac{d\omega }{\mathrm{dt}}-K\cos\theta \right]$$

Equation (A24) is an ordinary differential equation (ODE) governing the motion of droplet down the inclined wedge fixture having two hydrophobic plates with different wetting states. Since the dependent variable of interest is droplet angular velocity (ω), $v_{y}$ and can be eliminated from Equation (A24) via using Equations (A11) and Equation (A12). Hence, dvydt=gsinα1+25sin2(ϕ2)  and vy=gt sinα1+25sin2(ϕ2) are substituted in Equation (A24) and rearrangement results in: sinθ=vyRωsinϕ2=gt sinαRωsinϕ2(1+25sin2(ϕ2)) and cosθ=(1−(1+25sin2(ϕ2))2R2ω2sin2(ϕ2)).

The final arrangement gives rise to:

(A25) dωdt=a2tR2ωsin2(ϕ2)2 +K(1−a2t2R2ω2sin2(ϕ2))

where, a is linear acceleration presented in Equation (A11) as $\frac{{\mathrm{dv}}_{y}}{\mathrm{dt}}$.

Introducing A=a2R2sin2(ϕ2), the governing equation of motion (ODE) can be obtained for ω in terms of f(K,R,α,ϕ,t). Hence, it yields:

(A26) $$\frac{d\omega }{\mathrm{dt}}=\frac{\mathrm{At}}{\omega }+K\sqrt{\left(1-\frac{{\mathrm{At}}^{2}}{\omega^{2}}\right)}$$

The resulting angular acceleration for rolling and spinning droplet is in a nonlinear ordinary differential form of the first order and it depends on inclination angle, wedge angle, wetting states of two hydrophobic surfaces, properties of droplet fluid, and size of the droplet.

The solution of non-linear ODE can be obtained via using Wolfram Alpha (Mathematica Program), which yields:

(A27) $$\omega \left(t\right)=\sqrt{t^{2}\left(A+K^{2}\right)+2{\mathrm{Kte}}^{c1}+e^{2c1}}$$

The imposing initial condition of ω(0)=0 (consideration of droplet is at rest in the wedge fixture initially), a specific solution can be obtained as:

(A28) $$\omega =\;t\sqrt{\left(A+K^{2}\right)}$$

The inclination of the spinning axis (θ) can be obtained as:

(A29) θ=sin−1(gt sinαRt(A+K2)sinϕ2(1+25sin2(ϕ2)))

Therefore, rolling (rotational) angular velocity $\omega_{x}\left(t,\;A,\;K,\;\alpha ,\;\phi ,\;R\right)$ can be written as:

(A30) ωx=ωsin(θ)=gt2 sinαRsinϕ2(1+25sin2(ϕ2))

Spinning angular velocity, $\omega_{z}\left(A,K,\;\phi ,\;R\right)$ can be expressed as:

(A31) ωz=(t2(A+K2)−(1+25sin2(ϕ2))2R2sin2(ϕ2))

It is worth to mention that spinning velocity ($\omega_{z}$) is a function of droplet fluid property, droplet size, hydrophobic surface wetting states, and the wedge fixture angle.

The constant K involves droplet properties and it can be determined. From mass and volume relation;

(A32) $$m=\frac{4{\rho \pi R}^{3}}{3}$$

and K yields:

(A33) $$K=\frac{F_{\mathrm{ad}1}R\cos\left(\frac{\phi }{2}\right)-F_{\mathrm{ad}2}R\cos\left(\frac{\phi }{2}\right)}{\frac{8{\rho \pi R}^{5}}{15}}$$

Adhesion forces; $F_{\mathrm{ad}1}$ and $F_{\mathrm{ad}2}$ can be evaluated from [32].:

(A34) $$F_{\mathrm{ad}1}=\frac{48}{\pi^{3}}{\gamma \mathrm{Rf}}_{1}\left(\cos\theta_{R1}-\cos\theta_{A1}\right)$$

and

(A35) $$F_{\mathrm{ad}2}=\frac{48}{\pi^{3}}{\gamma \mathrm{Rf}}_{2}\left(\cos\theta_{R2}-\cos\theta_{A2}\right)$$

Hence, K takes the form:

(A36) $$K=\frac{120\gamma \left[f_{1}\left(\cos\theta_{R1}-\cos\theta_{A1})-f_{2}(\cos\theta_{R2}-\cos\theta_{A2}\right)\right]}{{\rho \pi }^{3}\forall \cos\left(\frac{\phi }{2}\right)}$$

where, γ is the surface tension of droplet fluid. $\theta_{R1}$, $\theta_{A1}$, $\theta_{R2},{\;\mathrm{and}\;\theta }_{A2}$ are advancing and receding angles of the droplet at hydrophobic plate surface 1 and hydrophobic plate surface 2, respectively, f is the solid surface fraction, which is defined through the ratio of area covered by texture pillars over projection area of the textured surface, R is the radius of droplet before deformation, ∀ is droplet volume, ω is the angular velocity of droplet down the inclined wedge fixture, ρ is the density of droplet fluid, ϕ is wedge angle of the fixture, which is formed by the two hydrophobic plate surfaces.
